# Lectin staining and flow cytometry reveals female-induced sperm acrosome reaction and surface carbohydrate reorganization

**DOI:** 10.1038/srep15321

**Published:** 2015-10-16

**Authors:** Jukka Kekäläinen, Irma Larma, Matthew Linden, Jonathan P. Evans

**Affiliations:** 1 University of Western Australia, Centre for Evolutionary Biology, School of Animal Biology (M092), Crawley, Australia; 2 University of Eastern Finland, Department of Biology, Joensuu, Finland; 3 University of Western Australia, Harry Perkins Institute of Medical Research, Centre for Microscopy, Characterization and Analysis, Crawley, Australia

## Abstract

All cells are covered by glycans, an individually unique layer of oligo- and polysaccharides that are critical moderators of self-recognition and other cellular-level interactions (e.g. fertilization). The functional similarity between these processes suggests that gamete surface glycans may also have an important, but currently overlooked, role in sexual selection. Here we develop a user-friendly methodological approach designed to facilitate future tests of this possibility. Our proposed method is based on flow cytometric quantification of female-induced sperm acrosome reaction and sperm surface glycan modifications in the Mediterranean mussel *Mytilus galloprovincialis*. In this species, as with many other taxa, eggs release water-soluble factors that attract conspecific sperm (chemoattraction) and promote potentially measurable changes in sperm behavior and physiology. We demonstrate that flow cytometry is able to identify sperm from other seawater particles as well as accurately measure both acrosome reaction and structural modifications in sperm glycans. This methodological approach can increase our understanding of chemically-moderated gamete-level interactions and individual-specific gamete recognition in *Mytilus* sp. and other taxa with similar, easily identifiable acrosome structure. Our approach is also likely to be applicable to several other species, since carbohydrate-mediated cellular-level interactions between gametes are universal among externally and internally fertilizing species.

Pre-fertilization physical interactions between gametes are mediated by various carbohydrates and proteins found on the surfaces of the sperm and eggs^1^,^2^,^3^,^4^,^5^,^6^. However, gametes have also been shown to communicate prior to physical contact via soluble egg compounds, which attract sperm cells towards unfertilized eggs (sperm chemotaxis^7^,^8^,^9^) and trigger changes in sperm biochemistry and physiology, including capacitation (sperm activation)^10^,^11^ and the acrosome reaction (release of proteolytic enzymes)^11^,^12^. Accordingly, chemical signaling between gametes clearly has a strong naturally selected function in sexual reproduction (fertilization)^13^. Interestingly, recent work has revealed that chemically moderated gamete interactions play the additional role of facilitating individual-specific gamete ‘preferences’, such that sperm from individual males consistently swim towards (and fertilize) the eggs of certain females^14^,^15^. As such, chemical communication is likely to play an important role in post-mating sexual selection (e.g. cryptic female choice^16^,^17^,^18^,^19^,^20^,^21^). However, understanding the mechanisms underlying such individual-specific gamete interactions remains a major challenge in evolutionary biology^22^.

The surfaces of all cells (including gametes) are covered by glycans – oligo- and polysaccharide molecules attached to cell membrane proteins and lipids^23^,^24^. Glycans therefore act as a first interface between cells and their environment. Glycans also have extraordinary structural diversity, which may have evolved as a direct consequence of their key role in pathogen recognition^24^ and many other cellular interactions, including fertilization^3^,^4^,^25^,^26^,^27^,^28^. The high diversity of glycans allows highly specific molecular-level interactions between cells, since each individual organism exhibits unique glycan patterns that distinguish them (“self”) from all other “non-self” organisms (self-recognition^24^). Prior to fertilization the sperm plasma membrane undergoes remarkable structural re-organization, including modifications in the appearance and structure of glycoproteins and other glycoconjugates^25^,^29^,^30^,^31^,^32^. These pre-fertilization sperm glycan modifications are critical regulators of sperm motility and essential determinants of sperm capacitation, the acrosome reaction and ultimately successful fertilization^25^,^29^,^33^,^34^. It has also been shown that carbohydrate-enriched sperm membrane proteins may play an important role in regulating intracellular Ca^2+^ concentration^2^,^35^, which in turn is an important factor regulating sperm chemoattraction towards unfertilized eggs^36^.

Taken together, the evidence that glycans exhibit striking structural complexity and play a key role both in cellular-level self-recognition and fertilization^37^ (self-recognition in marine invertebrates: see e.g. Smith *et al.*^38^), suggests that glycans may also mediate individual-specific gamete preferences during the fertilization process. To best of our knowledge, however, only one study has tested this possibility in mice^23^. Interestingly, Ghaderi *et al.*^23^ reported that sperm surface carbohydrates invoke an immune response in females which leads to reproductive incompatibility between particular males and females that have mismatched cell surface glycans^23^. Unfortunately, due to the structural complexity of glycans, and technological limitations of glycomics, molecular-level description of biologically important structural and temporal changes in cell surface glycans are currently largely unfeasible^39^,^40^. However, some current glycobiological tools, including lectins (carbohydrate-binding proteins), can offer powerful methodological approaches that can significantly increase our current understanding of the structure and function of the cell surface carbohydrates, especially in intact cells or tissues^39^,^41^.

The Mediterranean mussel *Mytilus galloprovincialis* provides a useful model system for exploring cellular-level interactions underlying individual-specific gamete recognition. *Mytilus galloprovincialis* is a sessile, sexually monomorphic broadcast spawning marine invertebrate distributed among temperate regions of the Northern and Southern Hemispheres. As with many other broadcast spawners, behavioral components of fertilization are absent in *M. galloprovincialis* and successful fertilization is likely to depend exclusively on gamete-level interactions that determine whether sperm from a particular male are successful in fertilizing eggs from a given female^22^. Two recent studies on *M. galloprovincialis* provide intriguing evidence that fertilization is moderated by the differential effects of egg-derived soluble factors on sperm chemoattraction, and consequently that sperm from individual males exhibit differential but consistent ‘preferences’ for eggs from particular females^14^,^15^. However, in *M. galloprovincialis*, as in numerous other species in which fertilization biases are contingent on female-moderated effects (cryptic female choice), the mechanistic basis for such sperm-egg interactions at fertilization remains virtually unknown.

In this study we develop a user-friendly methodological approach that is ultimately designed to offer insights into the role of gamete-surface glycans in moderating fertilization dynamics in *M. galloprovincialis*. It is known that in *Mytilus*, sperm undergo an acrosome reaction when mixed with egg water, which explains why the addition of egg water just prior to insemination significantly increases fertilization rates^42^,^43^. Due to the large size of the intact *Mytilus* acrosome, the acrosome reaction of these species can be observed under a microscope^44^. However, potentially more reproducible high throughput methods (such as flow cytometry) for observing the acrosome reaction have not yet been developed or tested for these species. Here we develop a flow cytometric approach that can be used to quantify female (egg water) induced acrosome reaction and associated sperm surface glycan reorganizations (visualized by lectins).

## Results

### Identification and gating of the sperm in flow cytometry

**Figure 1:**
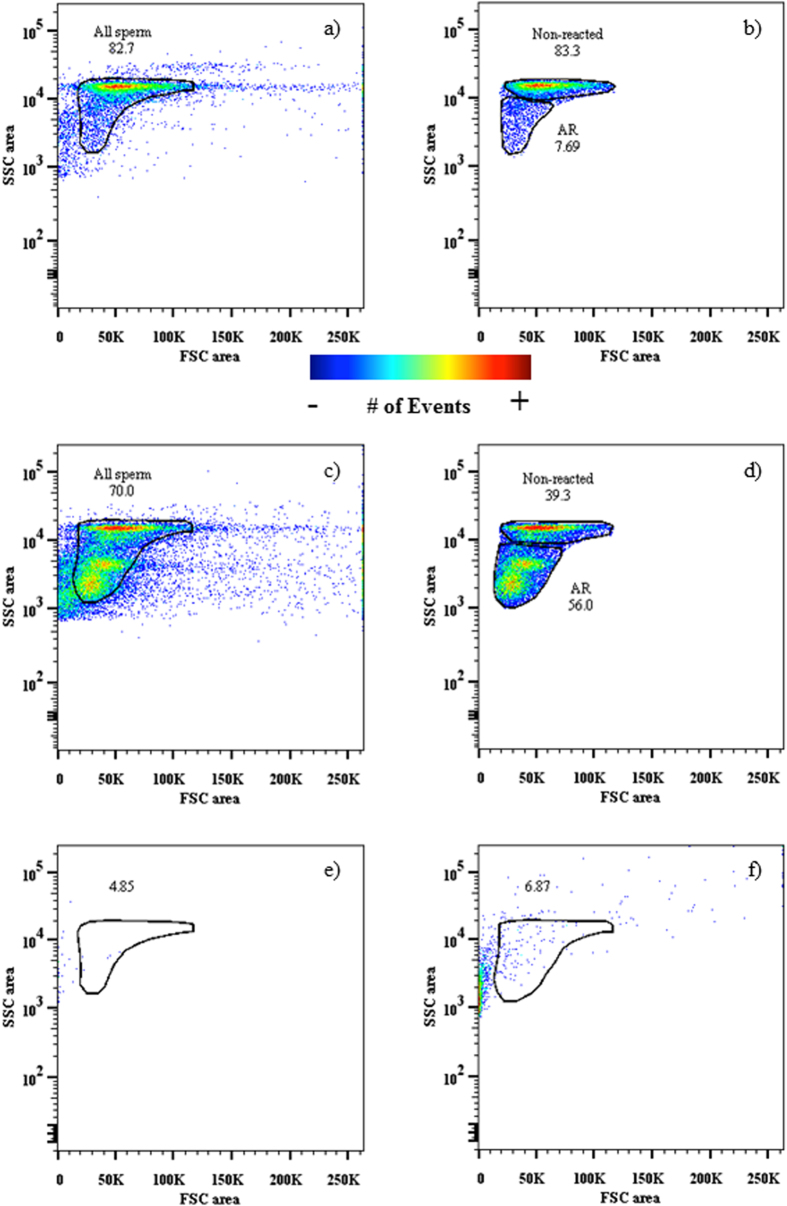
Flow cytometer gating strategy for sperm treated with seawater (control: (**a,b**)) and egg water (seawater + egg water, 1:1: (**c,d**)). Gating was based on FSC and SSC area of the sperm, which separated sperm from the other particles of the seawater (**e**) and egg water (**f**). The sub-population “Non-reacted” represents sperm with intact acrosomes, while the sub-population “AR” represents acrosome-reacted sperm. Numbers indicate proportions (%) of gated events of all events.

We first confirmed that flow cytometry is capable of separating sperm from other seawater particles. We found that forward scatter (FCS, particle size) and side scatter (SSC, granularity/complexity of the particle) characteristics (see methods) of both pure seawater (no sperm present) and egg water samples (*n* = 5, in both groups) were clearly different than that of sperm: both water samples contained only a small number of particles within the gated sperm population (SW: < 0.001%; EW < 1.0%) (Fig. 1). Therefore, any effect of non-sperm particles in potentially biasing our results is likely to be negligible. Egg water treatment (acrosome reaction) increased the relative proportion of small (FSC < 20 000) particles (Fig. 1c), which were largely absent when most of the sperm had intact acrosomes (Fig. 1a). This indicates that this group of particles may represent fragments of broken acrosomes or other particles released by eggs or sperm.

### Acrosome reaction in sea water vs. fresh egg water

**Figure 2:**
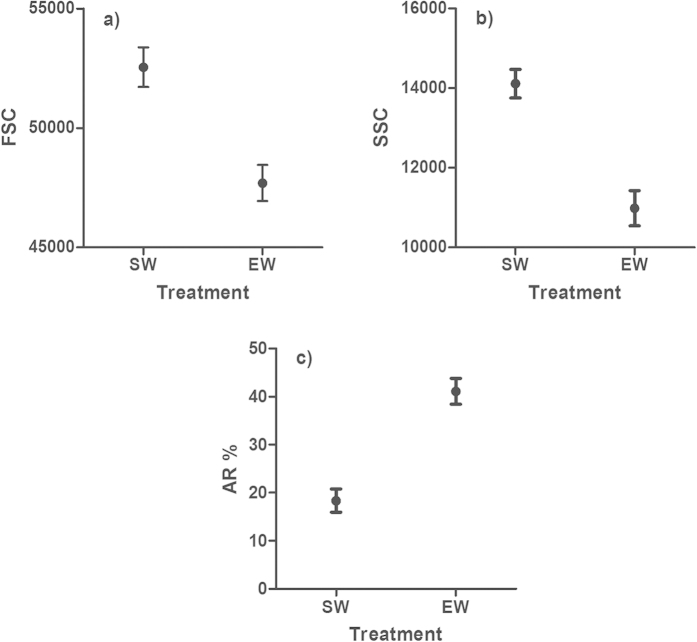
Measured flow cytometer variables for sperm acrosome reaction. Dots represent mean (±s.e.) values of forward scatter (FSC) area (**a**), side scatter (SSC) area (**b**) and the proportion of sperm in the sub-population (see Fig. 1) “acrosome-reacted sperm” (**c**) in sea water (SW) and egg water (EW) treatments. *N* = 31 in all cases.

Both microscopy (Linear mixed model, LMM, d.f. = 13, *t* = 3.206, *P* = 0.007) and flow cytometry analyses (LMM, d.f. = 30, *t* = −8.151, *P* < 0.001) showed increased proportion of acrosome reacted sperm cells (PC score, Fig. 2) in fresh egg water compared to the seawater (control). In addition, the proportion of acrosome reacted sperm cells (sub-population “AR” in Fig. 1) determined by flow cytometry predicted the proportion of confirmed acrosome reacted sperm in the microscopic analyses (LMM, d.f. = 23.1, *t* = −3.429, *P* = 0.002, Pearson, *r* = 0.667, *P* < 0.001).

### Microscopic analysis of lectin binding

**Figure 3:**
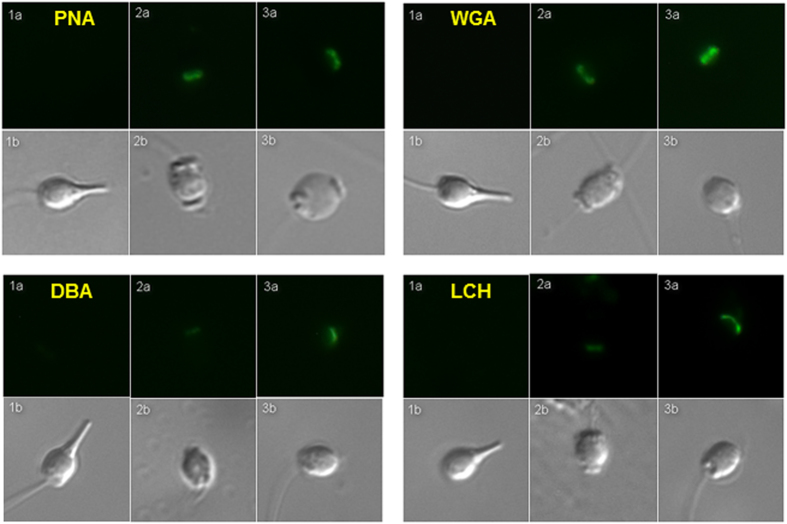
Fluorescent (**a**) and differential interference contrast (**b**) micrographs of sperm labeled with four lectins. Acrosome-intact (non-reacted) sperm are shown on the left (1), partially-reacted sperm in the middle (2) and fully acrosome-reacted sperm on the right (3).

The proportion of lectin-labeled (percentage of fluorescent) sperm cells was higher in the egg water than in the seawater (control) samples for DBA and LCH (Paired t-test, *P* < 0.001 and *P* = 0.027, respectively). However, this difference was not apparent for WGA and PNA (*P* > 0.05). This is likely to be attributable to the higher binding affinity of WGA and PNA on sperm, meaning that most of the WGA- and PNA-labeled sperm produced visible fluorescent signals, irrespective of the treatment. By contrast, unstained sperm were common in the DBA and LCH labeled samples (J.K., personal observations). Thus, although the intensity of fluorescence clearly differed between treatments, the proportion of WGA- and PNA-stained sperm cannot explain this difference. Microscopic examinations also revealed that all four lectins bind on the acrosomal region of the acrosome-reacted and partially-reacted sperm, but not on the sperm with intact acrosomes (Fig. 3).

### Flow cytometric analysis of lectin binding

**Figure 4:**
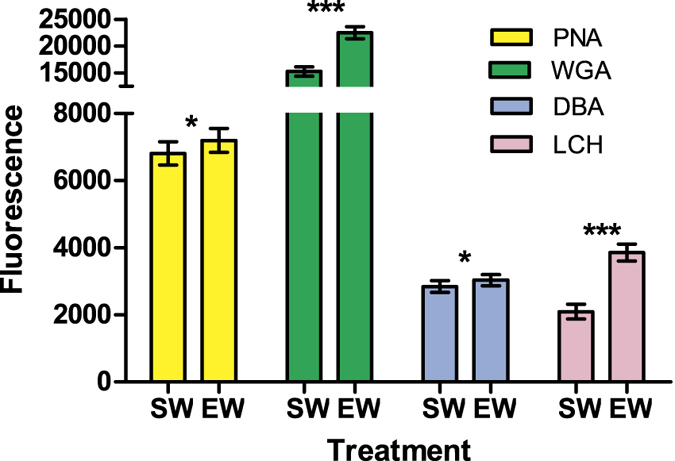
Sperm lectin binding measured by flow cytometer. Bars represent mean (±s.e.) fluorescence intensity of four lectins (PNA, WGA, DBA and LCH) in sperm samples treated with sea water (“SW”) or fresh egg water (“EW”). Asterisks indicate statistically significant differences between treatments (*P < 0.05; ***P < 0.001). Note differential scaling of the top and bottom segments of y-axis.

**Figure 5:**
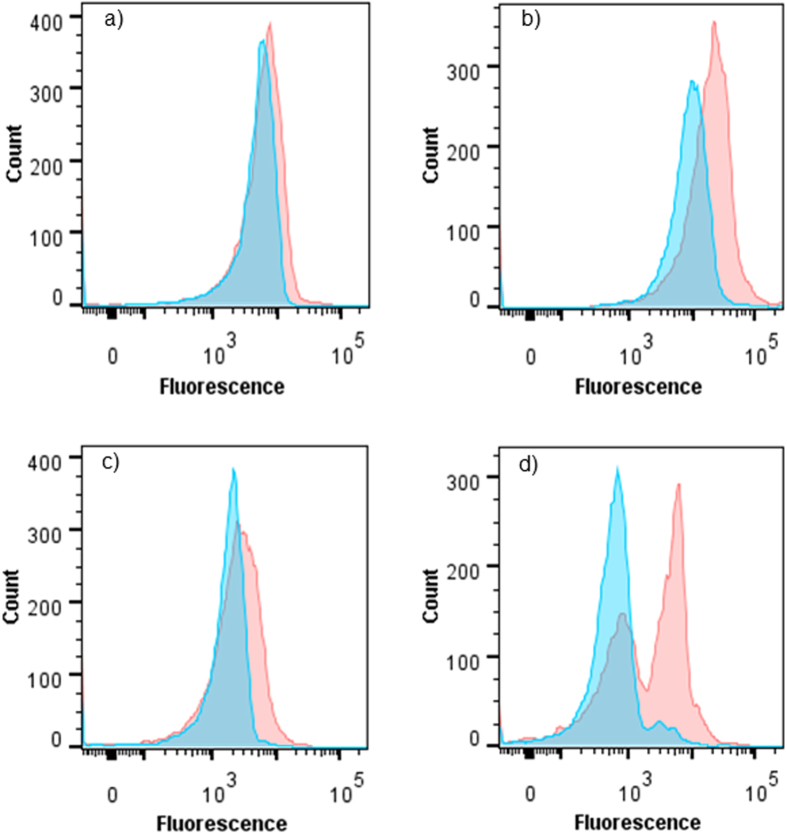
Fluorescent intensity of sperm labeled with PNA (**a**), WGA (**b**), DBA (**c**) and LCH (**d**). Blue colour = Sea water − treated sperm; Red colour = Egg water − treated sperm.

The mean fluorescence intensity of sperm was higher in sperm samples treated with egg water compared to those treated with seawater (LMM, d.f. = 203.6, *t* = 3.457, *P* < 0.001, Figs 4 and 5), and the level of fluorescence intensity differed between lectins (d.f. = 203.7, *t* = −12.147, *P* < 0.001). No statistically significant interaction was found between treatment and lectins (d.f. = 202.6, *t* = 1.912, *P* = 0.06, removed from the final model), suggesting that all four lectins had higher affinity (i.e. higher fluorescence) with sperm when the sample was treated with the egg water (Fig. 4). This finding is also supported by separate analyses of mean fluorescence difference for each lectin (*P* < 0.05 in all cases).

### Acrosome reaction and lectin binding in fresh and frozen egg water

The proportion of acrosome reacted sperm cells (LMM, d.f. = 15, *t* = 1.082, *P* = 0.30) and the mean intensity of fluorescence did not differ between fresh and frozen egg water samples (LMM, d.f. = 117, *t* = −0.332, *P* = 0.74), although consistent with our previous findings the fluorescence intensity differed among lectins (d.f. = 117, *t* = −7.172, *P* < 0.001). No interaction was found between treatment and lectins (d.f. = 116, *t* = −0.245, *P* = 0.81, removed from the final model), indicating that both egg water treatments had a similar influence on the binding capacity of the four lectins.

### Specificity of lectin binding

**Figure 6:**
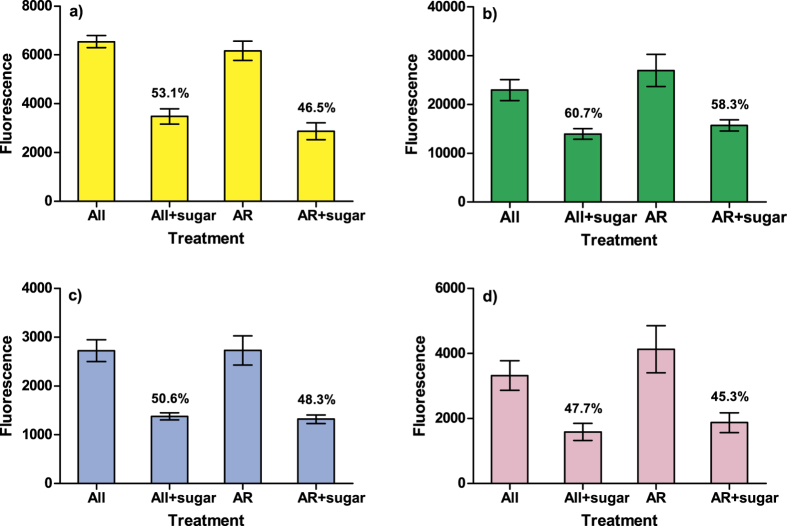
Specificity of lectin binding. Bars represent mean (±s.e.) fluorescence of PNA (**a**), WGA (**b**), DBA (**c**) and LCH (**d**) in total (“All”) sperm and acrosome-reacted sperm population (“AR”) labeled with 10 μg/ml of lectins with or without pre-treatment of inhibiting sugars. Percentages indicate the relative proportion of fluorescence intensity of sugar-treated samples in relation sperm without pre-treatment.

The mean fluorescence intensity of sperm was significantly lower when lectins were treated with their inhibiting monosaccharide sugars (LMM, d.f. = 76, *t* = −3.767, *P* < 0.001), and this effect was similar for all lectins (lectin-sugar – interaction: d.f = 72 *t* = −0.293, *P* = 0.77). Overall, mean fluorescence intensity was on average ca. 50% lower in sugar treated samples than when sperm were labeled with untreated lectins (Fig. 6). The fluorescence intensity (±s.e.) of sperm increased on average 4.36-fold with 10-fold increase (5 to 50 μg/ml) in lectin concentration (Table 1). The microscopic analysis did not reveal any observable differences in the spatial staining pattern of the sperm treated with different lectin concentrations: In all concentrations, lectins were found to bind predominantly to the acrosomal region of the sperm; non-reacted sperm exhibited no observable lectin binding at any concentration.

## Discussion

The present study demonstrates that flow cytometry is capable of separating *M. galloprovincialis* sperm from other seawater particles and highlights its utility in identifying and quantifying the egg water – induced acrosome reaction and sperm surface glycan modifications. Overall, the method developed here can offer a significantly faster and more objective alternative to traditional microscope – based approaches for assessing such sperm membrane structural changes. All four lectins (PNA, WGA, DBA and LCH) were confirmed to bind to the acrosomal region of the fully and partially acrosome-reacted sperm, but not acrosome-intact sperm (as also demonstrated by McAnlis 2007, for PNA, WGA and LCH^45^). Egg water increased the intensity of this binding compared to filtered seawater (control treatment) in all four lectins, but the difference between treatments was greatest for WGA and LCH, suggesting that these two lectins may be the most suitable markers to study the egg-water induced changes in *M. galloprovincialis* sperm physiology. We also demonstrate that fresh and frozen egg water exhibit equal capacity in inducing both acrosome-reaction and sperm surface glycan modifications, which make this technique amenable e.g. to species in which simultaneous gamete collection is not possible.

The treatment of lectins with their inhibiting monosaccharide sugars (200 mM) significantly reduced lectin binding, but did not prevent it completely. However, increasing concentration to 500 mM did not reduce lectin binding any further (data not shown). The sensitivity of the fluorescent probes (fluorescein isothiocyanate, FITC) to high temperatures and changes in ionic composition prevented us from using alternative methods (i.e. heat or chemical denaturation of the lectins) to investigate specificity. Full saturation of the binding was not observed when lectin concentrations were increased, suggesting that all the lectin binding receptors were not saturated even in the highest concentration (50 μg/ml). This conclusion was supported by our microscopic examinations showing similar spatial (acrosomal region) binding pattern at all tested lectin concentrations. However, as we note above, it seems likely that binding by all four lectins was not completely specific to their target monosaccharides (see also Fallis *et al.*^46^ for similar finding in other mussel species). Such findings are perhaps not surprising given that the biological ligands of the lectins are more complex than single monosaccharides and lectins have significantly higher binding affinity on such complex carbohydrate structures^39^,^46^,^47^,^48^,^49^. Thus, the demonstration of truly non-specific binding would require the use of more complex inhibiting carbohydrates. This conclusion is also supported by the fact that both the overall binding affinity (fluorescence intensity) and monosaccharide non-specificity was found to be highest for WGA, a lectin which is known to have especially broad carbohydrate specificity^50^. Furthermore, the high salt concentration of the seawater can prevent binding on structurally simple monosaccharides (such as inhibiting hapten sugars), but may not severely impede binding to more complex sperm surface carbohydrates as these bindings are structurally stronger^47^,^48^. Taken together, although we cannot exclude the possibility of some non-specific binding, it is likely that a large majority of the observed lectin binding was specific to their target glycan structures.

Our proposed method creates opportunities for shedding light on the mechanisms underlying differential sperm-egg interactions in *M. galloprovincialis*^14^,^15^. For example, this approach could be tailored readily to determine whether sperm acrosome reaction and glycan structural re-organizations exhibit differences between different male (sperm) - female (egg water) – combinations (i.e. male-by-female interaction). The use of factorial crosses involving multiple males and females offer promising tools to answer this question^22^. Such designs would make it possible to determine whether the acrosome reaction and associated glycan modifications (as determined through flow cytometry) depends on the specific combination of males and females present in each cross. We are currently pursuing such work in *M. galloprovincialis*, to test whether recently documented male-by-female interaction effects on fertilization, sperm chemotaxis and sperm behavior^14^,^15^ are directly attributable to demonstrated structural changes in sperm membrane. As such, the proposed methodological approach offers great promise to reveal the mechanisms underlying post-mating sexual selection (cryptic female choice) for genetically compatible sperm in this system.

Finally, the approaches advocated here are likely to be applicable to a broad range of taxa, especially species with analogous, easily identifiable acrosome structures^42^,^51^,^52^ or those in which the binding of fluorescently labeled lectins have been shown to signal the sperm’s acrosomal status^30^,^46^,^53^,^54^,^55^,^56^. This, in turn, opens interesting possibilities for testing the generality of our findings across a broader range of taxa, and extending this work to focus on the mechanistic basis for post-mating sexual selection in other systems. For example, in addition to egg-derived soluble factors (chemoattractants), a number of other female-derived fluids trigger the acrosome reaction, including follicular and oviductal (or ovarian) fluid in humans^57^, other mammals^58^ as well as many externally fertilizing species, such as amphibians^59^. Consequently, our proposed approaches may be more widely applicable than originally envisaged, allowing researchers to explore a range of potential mechanisms underlying female-moderated control of fertilization.

## Methods

### Collection, maintenance and spawning of the mussels

Mussels were collected by hand from Woodman Point Jetty, Western Australia (32°14’04”S, 115°76’25”E) and transported to the University of Western Australia (UWA, Crawley campus, Perth). Mussels were maintained in recirculating filtered seawater aquaria (water temperature +18 °C) until required. Prior to each experiment gamete release was induced by transferring the mussels to 60 × 37 × 37 cm plastic boxes containing seawater (to approx. 3 cm depth) preheated to 26 °C^14^,^60^. When an individual commenced spawning, we immediately washed it in clean, filtered seawater (to prevent contamination by gametes from other individuals) and placed it in an individual cup containing ca. 30 ml of filtered seawater, where mussels continued spawning.

### Collection of the egg water and sperm

Females (*n* = 31) were induced to spawn as described above and left to release eggs for 60 minutes. The eggs were then counted and the volume of filtered seawater was adjusted to achieve a final concentration of 12 000 eggs ml^−1^. The final egg-seawater –dilution was filtered with Whatman filter paper (11 μm retention size) to remove eggs and other particles from the filtered water (hereafter referred as ‘egg water’). The resultant egg water was subsequently mixed with sperm either fresh (hereafter ‘fresh egg water’) or after freezing the sample (‘frozen egg water’). The samples designated as frozen egg water (*n* = 16) were placed in a −80 °C freezer for 60 min (until thoroughly frozen) and then allowed to thaw to +20 °C. To obtain sperm samples, 31 males were left in their individual cups until they had spawned an excess number of sperm. Sperm density was then adjusted to 20 × 10^6^ sperm ml^−1^ by diluting the sperm solution with filtered seawater.

### Experimental treatments and lectin staining of the sperm

The sperm from each male was divided into 10 separate 250 μl aliquots. Five sperm aliquots were then treated with 250 μl of egg water (fresh or frozen) and five aliquots were mixed with 250 μl of filtered seawater (controls). The seawater samples used for the control treatments were filtered with Whatman filter paper, as described above. After establishing these treatments, four of the sperm samples from each group (seawater and egg water) were labeled with four Fluorescein Isothiocyanate (FITC) – labeled lectins (Vector Laboratories, Inc., Burlingame, CA, USA): Peanut agglutinin (PNA), *Dolichos biflorus* agglutinin (DBA), *Lens culinaris* agglutinin (LCH) and Wheat germ agglutinin (WGA). The remaining sperm sample from both groups was left unstained as a control. PNA, DBA and LCH belong to the Leguminosae (Legume) lectin family, predominantly found in the seeds of plants belonging to the Fabaceae (bean) family and that have the highest carbohydrate specificity on terminal galactose, terminal N-acetylgalactosamine and non-terminal mannose residues, respectively^61^. WGA belongs to the Gramineae (cereal) family and had highest specificity on terminal N-acetylglucosamine. All sperm samples (other than the unstained controls) were mixed with 10 μg/ml of their designated lectins, vortexed for 3 s and incubated for 30 min at +20 °C in the dark. The lectin-labeled sperm samples were then ‘washed’ by centrifuging the samples for 7 min in 700 × g and then re-suspending the resultant pellets in Whatman-paper filtered seawater. Since the fixation of sperm increases the permeability of cell membranes, all lectin labelings were conducted using live sperm, thus ensuring that only sperm surface carbohydrates were labeled during this procedure^46^. Sperm samples were always used within 3 h of spawning while egg water was always used within 24 h of collection.

### Specificity of lectin binding

To study the specificity of lecting binding, 10 μg/ml of each individual lectin were incubated in 200 mM hapten sugars for 60 min. After incubation, sperm samples were labeled with these sugar-treated lectins as described above. The following sugars (monosaccharides) were used as inhibitors: D-Galactose (for PNA), *N*-Acetyl-D-glucosamine (for WGA), N-Acetyl-D-galactosamine (for DBA) and Methyl α-D-mannopyranoside (for LCH). To further elucidate potential non-specific binding, we also performed a concentration series for each of the four lectins by labeling the sperm of five males with four different lectin concentrations (5, 10, 20 and 50 μg/ml). Then the fluorescence intensity differences of the sperm between these concentrations were compared with flow cytometer and spatial labeling pattern of the lectins at each concentration was studied under microscope (see below).

### Flow cytometry and data analysis

All sperm samples were analyzed by using a BD FACS Canto II digital flow cytometer and data were acquired with BD Diva software (BD Biosciences, San Jose, CA, USA). FITC labeled lectins were excited with a 488 nm air-cooled solid state 20 mW sapphire laser and fluorescent emission was collected by the detector with 530/30 band pass filter. The number of counted events (representing acrosome-intact sperm, see below) was set to 10,000 and events were then recorded at low flow rate (ca. 10 μl/min). Prior to data recording the stream was allowed to stabilize for 30 sec. Fluorescent emission was collected in the range of 515–545 nm (530/30 bandpass filter). Alignment and calibration checks of the instrument were performed daily using CS&T beads (BD Biosciences). FITC calibration beads (BD Biosciences) were also run prior to each experiment day to detect any instrument sensitivity drift. The resultant data were analyzed using FlowJo software v7.6.5 (Treestar, Ashland, OR, USA). The total sperm population was identified and gated based on characteristic voltage pulse area measurement of forward scatter (FSC: particle size) and side scatter (SSC: granularity/complexity of the particle) of the laser light (Fig. 1). The sperm population was then divided into two non-overlapping sub-populations representing acrosome reacted and non-reacted sperm, respectively. In order to confirm that the sperm gating strategy was capable of separating sperm from other particles that occur naturally in seawater and egg water, we also analyzed both of the water samples separately (i.e. without adding sperm, see Fig. 1e,f). Both samples were analyzed at low flow rate and recorded for 15 s, representing identical measurement conditions to those used for the actual sperm measurements. Finally, we determined mean values of FSC, SSC and FITC fluorescence intensity (i.e. the measure of binding affinities for the four lectins) for total, acrosome-reacted and non-reacted sperm populations. We also determined the proportion of acrosome-reacted cells in each sample: (number of reacted cells/number of all sperm) * 100. All flow cytometry analyses were conducted within 3 h of lectin staining.

### Microscopic analyses

In order to confirm that the two sperm sub-populations detected by flow cytometer actually represented acrosome reacted and non-reacted cells, we further determined the acrosome status of sperm for 14 males (representing a subset of all 31 males) using microscopy. Sperm samples were first analyzed using a flow cytometer as described above. We then determined the acrosome status of 100 (2 × 50) sperm cells under a microscope (800 × magnification). The proportion of acrosome-reacted cells in these samples was then compared to the proportion of these cells identified in the flow cytometer. We also tested whether the measured differences in lectin binding (i.e. fluorescence intensity) between egg water and control (sea water) treatments predict the proportion of lectin-labeled sperm cells in the microscopic analyses. To this end, both egg water and control samples (*n* = 6, in both groups) were labeled with 30 μg/ml of the above-mentioned four lectins. We then confirmed the lectin labeling status of the sperm and determined the proportion of lectin-labeled cells by counting 100 (2 × 50) cells under a fluorescent microscope (400 × magnification). The difference in the proportion of labeled cells between groups (egg water vs. control) was tested using paired t-tests. Finally, the spatial labeling pattern of the lectins on the sperm surface was studied with fluorescent and differential interference contrast microscopy (Zeiss Axioskop 2 Plus, 100 x oil immersion objective). Digital micrographs were captured using a Zeiss AxioCam MR and Axiovision software.

### Statistical analyses

To reduce the number of correlated variables in the flow cytometer data, we conducted principal component analyses (PCA) for three variables indicative of the acrosome reaction: (1) mean FSC and (2) mean SSC of all sperm; and (3) the proportion of acrosome reacted cells (Fig. 1). The PCA generated one principal component (with eigenvalue >1), which explained 84.9% of variation in these traits and was positively loaded by FSC and SSC (0.833, 0.959, respectively) and negatively by the proportion of acrosome reacted cells (−0.967). The effect of sperm treatment (seawater control vs. fresh or frozen egg water) on the sperm acrosome reaction was tested in linear mixed-effects models (LMM), where the PC-score for the acrosome reaction was fitted as the response variable, sperm treatment as a fixed effect and male ID (*n* = 31) as a random effect. When testing the association between putatively acrosome reacted cells (flow cytometer data) and confirmed acrosome-reacted cells (microscope samples, *n* = subsample of 14 out of 31 males) we used an otherwise identical model, but with the inclusion of the latter variable as an additional fixed effect in the model. The effect of sperm treatment on lectin binding (i.e. mean fluorescence intensity) was tested as above, but using lectin mean fluorescence intensity as a response variable and sperm treatment and lectin identity (as well as their interaction) as fixed effects. The effect of inhibiting sugars on lectin binding was tested in an otherwise identical model, but with sugar treatment included as a third fixed effect. All the model fits were verified graphically using Q-Q plots and residual plots. All presented *P*-values are from two-tailed tests with *α* = 0.05. Mixed model analyses were conducted using lmerTest package (version 2.0–20) in R (version 3.1.2).

## Additional Information

**How to cite this article**: Kekäläinen, J. *et al.* Lectin staining and flow cytometry reveals female-induced sperm acrosome reaction and surface carbohydrate reorganization. *Sci. Rep.* **5**, 15321; doi: 10.1038/srep15321 (2015).

## Figures and Tables

**Table 1:** Fluorescence intensity (±s.e.) of four lectins in four different labeling concentrations.

Lectin	5 μg/ml	s.e.	10 μg/ml	s.e.	20 μg/ml	s.e.	50 μg/ml	s.e	× increase
PNA	3 977	180	5 586	205	7 795	197	13 282	1 062	3.34
WGA	12 774	548	19 051	1 219	26 301	1 691	45 855	3 055	3.59
DBA	1 079	124	1 573	160	3 004	159	5 984	357	5.55
LCH	2 061	138	3 542	267	5 419	304	10 180	421	4.94
